# Should we use Palivizumab immunoprophylaxis for infants against respiratory syncytial virus? – a cost-utility analysis

**DOI:** 10.1186/s13584-018-0258-4

**Published:** 2018-12-17

**Authors:** Gary M. Ginsberg, Eli Somekh, Yechiel Schlesinger

**Affiliations:** 1 Department of Technology Assessment, Public Health Service, Ministry of Health, Jerusalem, Israel; 2 Department of Pediatrics, Wolfson Hospital, Holon, and Sackler School of Medicine, Tel Aviv University, Tel Aviv-Yafo, Israel; 3 European Paediatric Association/Union of National European Paediatric Societies and Associations, Berlin, Germany; 4 Department of Pediatrics, Shaarae Zedek Medical Center, Affiliated to the Hadassah-Hebrew University Medical School, Jerusalem, Israel

**Keywords:** Cost-utility analysis, RSV, Immunoprophylaxis

## Abstract

**Background:**

Passive immunization against RSV (Respiratory Syncytial Virus) is given in most western countries (including Israel) to infants of high risk groups such as premature babies, and infants with Congenital Heart Disease or Congenital Lung Disease. However, immunoprophylaxis costs are extremely high ($2800–$4200 per infant). Using cost-utility analysis criteria, we evaluate whether it is justified to expand, continue or restrict nationwide immunoprophylaxis using palivizumab of high risk infants against RSV.

**Methods:**

Epidemiological, demographic, health service utilisation and economic data were integrated from primary (National Hospitalization Data, etc.) and secondary data sources (ie: from published articles) into a spread-sheet to calculate the cost per averted disability-adjusted life year (DALY) of vaccinating various infant risk groups. Costs of intervention included antibody plus administration costs. Treatment savings and DALYs averted were estimated from applying vaccine efficacy data to relative risks of being hospitalised and treated for RSV, including possible long-term sequelae like asthma and wheezing.

**Results:**

For all the groups RSV immunoprophylaxis is clearly not cost effective as its cost per averted DALY exceeds the $105,986 guideline representing thrice the per capita Gross Domestic Product. Vaccine price would have to fall by 48.1% in order to justify vaccinating Congenital Heart Disease or Congenital Lung Disease risk groups respectively on pure cost-effectiveness grounds. For premature babies of < 29 weeks, 29–32 and 33–36 weeks gestation, decreases of 36.8%, 54.5% and 83.3% respectively in vaccine price are required.

**Conclusions:**

Based solely on cost-utility analysis, at current price levels it is difficult to justify the current indications for passive vaccination with Palivizumab against RSV. However, if the manufacturers would reduce the price by 54.5% then it would be cost-effective to vaccinate the Congenital Heart Disease or Congenital Lung Disease risk groups as well as premature babies born before the 33rd week of gestation.

## Key points

Question: Should we continue using Palivizumab immunoprophylaxis for at-risk infants against Respiratory Syncytial Virus?

Findings: A cost-utility analysis which modelled the costs, resultant treatment savings and improvements in quality of life as a result of decreased morbidity from passive immunization, found for all risk-groups that RSV immunoprophylaxis is clearly not cost effective, unless vaccine prices fall considerably.

Meaning: Based solely on cost-utility analysis, at current price levels, it is difficult to justify the current indications for immunoprophylaxis against RSV.

## Background

In infants and young children the most common cause of severe lower respiratory tract disease is Respiratory syncytial virus (RSV). Most new-borns are infected before they are one year old, and virtually everyone gets an RSV infection by the age of two [1].

In Israel (Population 8.75 million [2]), RSV accounts for thousands of hospitalization days annually in children under two years old. The almost solitary identified chronic sequelae are possibly wheezing and asthma.

Since the disease course in high risk children is much more severe, and since no active vaccine is available, passive immunization with five sequential monthly injections of anti-RSV monoclonal antibodies (Palivizumab) is given during the RSV season (November – March). This schedule has proven to decrease hospitalization in high risk groups [3].

An RSV passive immunoprophylaxis course (costing around $6,300) is over a hundred times more expensive than courses of prophylaxis in the form of vaccinations against other infectious diseases such as measles, mumps, rubella, polio, diphtheria, pertussis, hepatitis or Haemophilus influenzae type B.

In 2001, despite its extremely high costs, passive vaccination using RSV was introduced in the high risk group of extreme premature babies (<30 Gestational Age in Weeks [GAW]), without any prior evaluation based on cost-effectiveness analysis. During the following years the indications for the passive vaccination were steadily expanded to include older premature babies. Currently, immunoprophylaxis is provided to infants with <35 GAW, as well as to infants at high risk such as those with CHD (Congenital Heart Disease) or CLD (Congenital Lung Disease), again with no underpinning cost-effectiveness analysis.

However, recently the American Academy of Pediatrics (AAP) narrowed the indication to those born with <29 GAW [3]. In response, the Israeli Association of Pediatrics decided to examine the application of restricting the guidelines for RSV immunoprophylaxis in Israel. An important component of this decision, although not the sole one, is a cost utility analysis.

Therefore we carried out a cost utility analysis of passive immunization with palmivizumab against RSV to see if the DALY (Disability Adjusted life Year) gains justify the high RSV immunoprophylactic costs in various at-risk groups.

## Methods

### Cost-utility analysis: Basic model

A Microsoft Excel spread-sheet model was constructed, incorporating vaccine efficacy, epidemiological, health service utilization, demographic and economic data (listed with sources in Table 4 in Appendix 1). The effect of vaccinations against RSV was modelled on incidence, chronic sequelae and mortality over a 100 year time horizon as is standard practice in order to capture the full effects of the intervention. The cost utility ratio calculated the net costs per averted Disability Adjusted Life Year (DALY) added as a result of passive immunization against RSV by means of palivizumab, using the formula:

$$ \mathrm{Net}\ \mathrm{Costs}\ \mathrm{per}\ \mathrm{a}\mathrm{verted}\ \mathrm{DALY}=\frac{\mathrm{Costs}\ \mathrm{of}\ \mathrm{immunoprophylaxis}-\mathrm{Savings}\ \mathrm{in}\ \mathrm{treating}\ \mathrm{RSV}\ \mathrm{a}\mathrm{nd}\ \mathrm{chronic}\ \mathrm{effects}}{\mathrm{Increase}\ \mathrm{in}\ \mathrm{DALY}\mathrm{s}\ \mathrm{a}\mathrm{s}\ \mathrm{a}\ \mathrm{result}\ \mathrm{of}\ \mathrm{decreased}\ \mathrm{mortality}\ \mathrm{and}\ \mathrm{morbidity}} $$

Costs are viewed from a societal perspective at mid-2015 price levels at the average annual exchange rate of 3.89 shekels to the US dollar [4]. Besides direct health service costs, we also included from a social perspective, costs due to work absences and transport costs to receive treatment. All future costs and DALYs were discounted at an annual rate of 3% as is the standard practice in Israel. While DALYs averted from reduced caregiver burden were available, data on out-of-pocket expenses was however not available.

The cost-utility ratios of immunoprophylaxis for the following various risk groups was calculated:-

- Congenital Heart Disease (CHD)
- Congenital Lung Disease (CLD)
- Prematures under 29 weeks gestation
- Prematures 29–32 weeks gestation
- Prematures 33–36 weeks gestation
- Not a member of any of the above risk groups

Evidence from studies relating to Bronchopulmonary Dysplasia (BPD) were included under the category of Congenital Lung Disease (CLD).

### Decision rules

Taking into account the resources available in Israel, an intervention was defined as being very cost-effective and cost-effective if the cost per averted DALY is less than the per capita GDP (gross Domestic product) of $35,329 in 2015 [2, 4] or between 1 and 3 times the per capita GDP ($35,329 – $105,987) respectively. If the cost per averted DALY is more than three times the GDP per capita ($105,987) then the intervention was regarded as not being cost-effective [5].

### Immunoprophylaxis

We assumed a five dose passive immunoprophylaxis schedule, using palivizumab, in which there would be a take up of 4.90 (for CLD) and 4.93 shots (for others), which was achieved in the clinical trials of palivizimab and hence influenced the overall effectiveness of the immunoprophylaxis schedule. Data from the “IMPACT” study [6] led us to assume there were no significant adverse palvizimab related events apart from minor effects. Immunoprophylaxis wastage was assumed to fall from 5.8% levels in 2008 to around 3.3% based on the implementation of improved delivery systems [7].

### Intervention costs

We used the current vaccine price, of $520 and $957 for 50 mg and 100 mg vials respectively, as a baseline price (excluding Value Added Tax as this is just a transfer payment). The unit immunoprophylaxis costs were applied to the average age-specific weights of the immunized children. Since at each point of the immunoprophylaxis schedule the infant received no other concurrent vaccinations, we included costs arising from transport and work losses. Provision was also made for treatment costs, transport costs and work losses arising from the visits to health service providers for minor side effects from the palivizimab passive immunization. Also included were the costs (and DALY losses) of long-term chronic sequelae from RSV from increased incidence of asthma and a more controversial possible increased incidence of wheezing.

Immunoprophylactic efficacy and its impact on hospitalizations and mortality from RSV by the risk groups were obtained by combining data from the literature (Table 4 in Appendix 1). Interpolations and extrapolations were extensively used due to the lack of homogeneity in reporting results by age and gestational age groups. Hospitalization rates and data on lengths of stay on account of RSV in Israel were based on data from the Ministry of Health’s National Hospitalization data base while mortality data was based on the National Deaths Registry (Personal Communication Ziona Haklaii and Nehama Goldberger).

Besides confirmed cases (of pneumonia and bronchiolitis) caused by RSV, we estimated that 13.2% [8–10] of hospitalizations recorded with an Otitis Media diagnosis were caused by RSV, and similarly that RSV was responsible for 40% of cases [11] of acute bronchitis (AB) recorded as being of unknown origin.

### Treatment costs

Acute care costs were calculated by multiplying the expected number of hospitalization days or visits by the unit costs of the respective ambulatory (ie: family practitioner and out-patient visits), emergency room and hospital services that were used.

Costs of sequelae (wheezing, asthma) were taken from the literature [12, 13] and adjusted to Israeli price levels, with 80% of costs (mainly labor costs) converted using purchasing power parity rates and the remaining 20% on exchange rates.

### Disability weights

Disability Weights (DW) associated with the pre-hospital, post-hospital and chronic phases (up to half a year) were obtained from the literature for both the patient [14]and the caregiver [14]. Additional DW for chronic sequelae after the chronic phase were based on five episodes a year of severe wheezing [13]and a similar number of annual asthma attacks [12]. All these DW were adjusted by the age specific DW of a healthy person.

### Averted DALY losses

Morbidity losses (with and without the intervention) were calculated from the product of changes in incidence (derived from the RR of the prophylaxis), the specific DW and the duration of the disability. Mortality losses were calculated by multiplying mortality rates (with and without the intervention – derived from the RR of the prophylaxis) by gender-specific the HALE (Healthy adjusted Life Expectancy) of the deceased.

Total DALY losses averted were based on the sum of the morbidity and mortality DALY losses, as a result of the passive vaccination lowering the incidence of RSV and Chronic sequelae. DALY losses resulting from caregiver burden were also included [14].

### Sensitivity analyses

One way sensitivity analyses were carried out by:- varying the number of hospitalizations attributable to RSV between 2,700-3,200 :- by excluding effects of long-term asthma, :- by varying the % of cases of otitis media and of Acute Bronchitis of unknown origin attributable to RSV: and finally by varying the values of the major input cost driver of immunoprophylaxis costs.

## Results

Because of the low prevalence of CHD and CLD in 2015 (0.16% of all births or 267 infants), passive immunization costs for these two risk groups would only total $1.67 million (Table 1). The costs of immunoprophylaxis of premature babies or children not at risk are considerably higher, being $83 and $1,037 million respectively. However, decisions should obviously not be made on the basis of cost alone and this justifies our cost-utility analysis that combines economic with epidemiologic data.

**Table 1:** Cost of providing RSV immunoprophylaxis to Infants by Risk Group (assuming 100% coverage)

Risk group	% of births	Births in 2015	Vaccination Cost USD
CHD	0.03%	50	314,057
CLD	0.12%	217	1,360,912
<29	0.42%	753	4,731,498
29–32	0.88%	1570	9,862,078
33–36	6.13%	10,956	68,814,325
No risk	92.42%	165,177	1,037,499,916

In our baseline situation, RSV caused approximately 2,945 hospitalizations each year in under two years of age babies. For all the risk groups and hospitalization ranges, even when the long-term effects of Asthma are included, passive immunization against RSV is clearly not cost effective as its cost per DALY is well in excess of the $105,986 guideline (Table 2). Of particular interest is the 29-32 GAW infants as the AAP does not recommend providing immunoprophylaxis to this age group. For this group the cost per DALY ratio is around ten times the GNP per capita level in Israel, meaning that giving pulmizamub to this group is clearly not justified on grounds of cost effectiveness, all the more so on those with 33-36 GAW (Table 2).

**Table 2:** Cost-utility ratios of providing RSV immunoprophylaxis to infants by risk group and by annual hospitalizations in children aged 0–2 years old (a)

Risk Group	Annual hospitalizations in children aged 0–2 years		
	2700	2945	3200
	Cost per DALY including asthma	Cost per DALY including asthma	Cost per DALY including asthma
CHD	$223,687	$218,968	$214,347
CLD	$303,658	$287,057	$268,242
<29	$246,594	$226,900	$208,765
29–32	$369,551	$347,593	$327,141
33–36	$1,211,273	$1,149,584	$1,092,860
No risk	$3,217,414	$3,023,294	$2,849,644

^a^cost-effectiveness threshold is $105,986 per averted DALY

RSV incidence would have to increase by between 56%-424% (depending on the at-risk group), to between 4,581-15,457 annual attributable hospitalizations in order that immunoprophylaxis would become cost- effective to specific at-risk groups (Table 2). Even if three-quarters of all the otitis media and unknown AB hospitalizations were attributable to RSV (instead of the estimated 13.2% and 40% respectively), this would only amount to 4,452 hospitalizations annually that could be attributable to RSV.

In the baseline situation, there would have to be a decrease in vaccine price of around 48% in order to justify passively immunizing CHD and CLD risk groups on pure cost-effectiveness grounds (Table 3). For premature babies of <29 weeks, 29-32 and 33-36 weeks gestation, decreases of 36.8%, 54.5% and 83.3% respectively in vaccine price would be required. Omission of long-term asthma effects, results in even higher cost per DALY ratios (Table 3) and even lower vaccine prices required to attain cost-effectiveness.

**Table 3:** Vaccine prices required to achieve cost-effectiveness ^a^

Risk group	Cost per DALY (excluding asthma)	Cost per DALY (including asthma)	Palivizumab price to achieve cost-Effectiveness (including asthma)		% decrease in Palivizumab Price
			50 mg Vial ^b^	100 mg Vial ^c^	
CHD	$266,020	$218,968	$278	$512	46.5%
CLD	$472,139	$287,057	$270	$497	48.1%
<29	$585,537	$226,900	$329	$605	36.8%
29–32	$685,961	$347,593	$237	$436	54.5%
33–36	$2,092,809	$1,149,584	$87	$160	83.3%
No risk	$7,503,953	$3,023,294	$31	$57	94.0%

^a^ based on threshold of $105,986 per averted DALY and 2945 annual hospitalizations in children under two

^b^ current price (excluding VAT) of $520 per vial

^c^ current price (excluding VAT) of $959 per vial

## Discussion

For all the groups and hospitalization ranges, passive immunoprophylaxis against RSV is clearly not cost effective. Based only on cost-effectiveness criteria, the current immunoprophylactic RSV policy should be stopped or modified and resources may be more efficaciously devoted to elsewhere in the health system.

However, due to their potentially harsh individual morbidity profiles, small numbers and hence far smaller budget impact, consideration could be given to continuing the passive immunization of infants belonging to the CHD and CLD risk groups, even at the current vaccine price levels.

If pressure could be asserted on the manufacturers to reduce the vaccine price by around 48.1% then it would be cost-effective to provide palvizimab only to the CHD and CLD risk groups. If the palvizimab price were to be reduced by 54.5% then it would also be cost-effective to provide passive immunization to the larger numbers of premature infants, born before the 33rd week.Our study’s finding that passive immunoprophylaxis of RSV is not cost-effective affirms the findings of numerous other studies in infant risk-groups [15–21] (Appendix 2). On the other hand, there are also many studies which reported that immunoprophylaxis was cost-effective [15, 19–32] (Appendix 2) or even cost-saving in some risk groups [19, 21, 33–37] (Appendix 2). Many studies [16, 17, 38–49] reported that Palivizumab infant immunoprophylactic costs exceeded the resultant savings in hospitalization costs (Appendix 2).

So is immunoprophylaxis cost-effective or not cost-effective (as our study shows)? Comparisons with studies in other countries have to be made with caution not only on account of differences in intervention costs, incidence rates, treatment modalities and costs, but also due to differing model specifications [50] and especially the funding source. Several studies tend towards showing lower net costs [51], especially those incorporating indirect costs due to valuing premature mortality by discounting future years productivity losses [15, 22–28] instead of using the method of friction costing [52] (which would be minimal in the event of infant or child deaths).

Our study was based on the acceptable practice of valuing premature morbidity using friction costing which take into account only the premature burial costs and marginal costs of possibly training a person to fill the job vacancy caused by the deceased person. The loss of the deceased person is captured mainly in terms of loss of disability adjusted life years as the monetary loss to society is minimal. We conclude that the major explanation of the existence of two large contradictory reports relating to the potential cost-effectiveness of immunoprophylaxis against RSV, is that the research results are dependent on the nature of the different funding sources.

An extensive Health Technology Assessment [53], which like this paper integrated data from many studies, concluded that prophylaxis with Palivizumab does not justify its cost. Nevertheless the study defined a few cost-effective groups (based on a threshold of 30,000 sterling, about 1.3 times the GDP per capita) such as in children under 6 weeks old at the start of the RSV season who had at least two risk factors and a < 25 GAW, or children with CHD or CLD under 6 weeks old and with < 25 GAW or < 29 GAW respectively.

The estimates in our study were fortunate to be based on quality of life estimates not only of the infant but also of the caregiver, a luxury not always enjoyed in most published cost utility analyses, outside the realm of dementia.

Costs per DALY could be considered to be overestimated since it could be possible to still further reduce vaccine wastage to around 1.5%, where large volumes are used [7]. On the other hand the cost utility ratio could be underestimated because we excluded the (negligible) room overheads for vaccination and publicity outreach costs.

In 2014, the risk groups of infants that received RSV immunoprophylaxis in Israel were expanded to include infants born prematurely between 33-34 weeks. As demonstrated in our study this decision (like the initial decision in 2001 to supply RSV vaccinations) was not based on any cost utility or cost effectiveness analyses.

A critical question is whether the introduction or expansion of medical technologies should be based only, mainly or partly on cost utility criteria. Pure cost utility based on comprehensive meta-analyses of available economic, medical and epidemiological information may dilute unwanted effects such as political pressure and lobbing by industry, by providing the decision makers with a clear “standard” for their decision.

On the other hand, there could be several reasons for the avoidance of using the gold-standard metric of cost-utility analysis such as the case of very rare diseases where the medical costs do not have significant economic impacts or societal consensus of providing priorities for specific groups such as neonates or pregnant women. However, even in these cases, cost utility analyses may provide alternatives for investments in these specific populations to get the best yield in terms of saving lives and reducing morbidities.

It is surely in the pharmaceutical industry's interest (and in the interest of free competition) that interventions should be objectively compared using cost-utility analysis (as per the National Institute for Clinical Excellence in the United Kingdom). The ministry could also use the results of cost-utility analyses to sometimes request decreases in unit costs so as to turn an intervention that is not cost-effective into one that is cost-effective or very cost-effective, as was achieved by the NHS regarding the recent meningococcal B vaccination in the United Kingdom [54].

We hope that this cost-utility analysis will provide the decision makers with a powerful and transparent tool to aid in logical decision for determining the extent of implementing technologies such as RSV prophylaxis. Only time will tell whether or not the results of our RSV analysis will modify the policy for the provision of immunoprophylaxis against RSV or another alternatives will be agreed on for improving the health of premature infants.

## Conclusions

Based on cost-utility analysis, at current price levels it is difficult to justify the current immunoprophylaxis program against RSV in Israel. However, if the manufacturers would reduce the price of the passive vaccine by 55.4% then it would be cost—effective to vaccinate the CHD and CLD risk groups as well as premature babies born before the 33rd week.

## Appendix 1

**Table 4:** Values and sources of model’s parameters

		Value		Source(s)
Epidemiologic				
Infants with BPD		0.037%		[55]
Infants with CHD		0.028%		[55]
Infants with CLD		0.084%		[55]
< 29 weeks gestation		0.42%		[56]
29–32 weeks gestation		0.88%		[56]
33–36 weeks gestation		6.13%		[56]
No Risk group		92.42%		[56]
	0–5	6–11	12–23	
	months	months	months	
Life Expectancy (years)	82.1	81.6	81.1	[4]
HALE (Health Adjusted Life Expectancy)	71.9	71.5	70.8	[a]
Discounted HALE	29.13	29.07	28.96	[a]
RSV Mortality per 1000 cases				
BPD	0.322	0.117	0.032	[6, 57–59]
CHD	0.658	0.330	0.045	[6, 57–59]
CLD	0.303	0.136	0.034	[6, 57–59]
<29	0.204	0.096	0.010	[6, 57–59]
29–32	0.186	0.018	0.004	[6, 57–59]
33–36	0.073	0.030	0.009	[6, 57–59]
No risk	0.013	0.004	0.001	[6, 57–59]
Immunoprophylaxis Efficacy against RSV Mortality				
BPD		0.17		[21]
CHD		0.22		[21]
CLD		0.17		[21]
< 29		0.73		[21]
29–32		0.76		[21]
33–36		0.79		[21]
No risk		0.81		[21]
Utilization				
*Hospitalizations in persons < 2 years old*				
(AB: Acute Broncholiosis)				
AB with RSV (466.11)		2011		[b]
% unknown diagnoses that are RSV		40%		[11]
AB unknown organisms: RSV (466.99)		843		[b]
RSV Pnuemonia (210.1)		50		[b]
% Otitis Media caused by RSV		15%		[8–10]
Otitis Media RSV		196		[b, c]
RSV < 2 yrs. old		3100		[b]
RSV 12–23 months		381		[b]
RSV 6–11 months		662		[b]
RSV < 6 months old		2057		[b]
Average length of stay aged 12–23 months		3.7	days	[b]
Average length of stay aged 6–11 months		3.6	days	[b]
Average length of stay < 6 months old		4.2	days	[b]
Relative Risks of Hospitalizations in Unvaccinated Children				
	0–5	6–11	12–23	
	months	months	months	
BPD	8.6	9.6	9.0	[58, 60]
CHD	2.7	4.2	2.0	[58, 60]
CLD	3.9	5.4	4.6	[58, 60]
<29	5.3	7.6	2.8	[58, 60]
29–32	3.4	6.2	5.4	[58, 60]
33–36	1.9	2.4	2.4	[58, 60]
No risk	1.0	1.0	1.0	
Average Lengths of Stay: General Wards (days) - Unvaccinated Children				
	0–5	6–11	12–23	
	months	months	months	
BPD	8.0	6.7	7.0	[6, 29, 61]
CHD	7.7	6.4	6.8	(b, [62])
CLD	7.9	6.6	6.9	(b, [62])
<29	3.7	3.0	3.2	(b, [62])
29–32	4.5	3.7	3.9	(b, [62])
33–36	5.8	4.8	5.0	(b, [62])
No risk	3.9	3.3	3.5	(b, [62])
Average Lengths of Stay ICU (days) - Unvaccinated Persons				
	0–23			
	months			
BPD	9.1			[59, 62]
CHD	10.1			[59, 62]
CLD	16.1			[59, 62]
<29	11.8			[59, 62]
29–32	12.3			[59, 62]
33–36	12.8			[59, 62]
No risk	6.7			[b]
Average Lengths of Stay General Wards (days) - Vaccinated Children				
	0–5	6–11	12–23	
	months	months	months	
BPD	7.0	5.8	6.1	(b,[6])
CHD	5.3	4.4	4.7	(b,[41, 61])
CLD	5.4	4.5	4.8	(b,[41, 61])
<29	2.8	2.3	2.4	(b, [63])
29–32	3.4	2.8	3.0	(b, [63])
33–36	4.4	3.6	3.8	(b, adjusted [63])
No risk	3.0	2.5	2.6	(b, adjusted [63])
Average Lengths of Stay ICU (days) - Vaccinated Persons				
	0–23			
	months			
BPD	3.9			(b,[6])
CHD	4.4			(b,[41, 61])
CLD	7.0			(b,[41, 61])
<29	5.9			(b, [63])
29–32	6.1			(b, [63])
33–36	6.4			(b, adjusted [63])
No risk	3.3			(b,adjusted [63])
Ratio of Ambulatory Visits to Hospitalizations				
	0–5	6–11	12–23	
	months	months	months	
BPD	3.7	26.0	16.9	[64–66]
CHD	3.7	26.0	16.9	[64–66]
CLD	3.7	26.0	16.9	[64–66]
<29	3.7	26.0	16.9	[64–67]
29–32	3.7	26.0	16.9	[64–67]
33–36	3.8	26.5	17.3	[64–67]
No risk	3.9	26.7	17.4	[64–67]
Ratio of Emergency Room Visits to Hospitalizations				
	0–5	6–11	12–23	
	months	months	months	
BPD	0.3	1.3	1.3	[64–66]
CHD	0.3	1.3	1.3	[64–66]
CLD	0.3	1.3	1.3	[64–66]
<29	0.3	1.3	1.3	[64–67]
29–32	0.4	1.4	1.4	[64–67]
33–36	0.5	1.9	1.9	[64–67]
No risk	0.5	2.0	2.0	[64–67]
RSV Sequelae				
Relative Risks by age for Asthma after Hospitalization for RSV				
	0–5	6–11	12–23	
	months	months	months	
BPD,CHD,CLD	1.03	1.05	1.17	assumed as for 29–32 weeks
<29	1.03	1.06	1.20	[68–72]
29–32	1.03	1.05	1.17	[68–72]
33–36	1.02	1.04	1.15	[68–73]
No risk	1.03	1.05	1.10	[68–73]
	3–10	11–20	21+	
	years	years	years	
BPD,CHD,CLD	1.11	1.09	1.14	assumed as for 29–32 weeks
<29	1.12	1.10	1.17	
29–32	1.11	1.09	1.14	
33–36	1.08	1.07	1.12	
No risk	1.09	1.09	1.15	
	[68, 71, 72, 74–81]	[68, 72, 78, 80]	[68, 72, 78, 80]	[age-specific]
Asthma attacks per year in not-fully controlled		5.0		Assumption
Asthma cases fully controlled		50%		Assumption
Severe Wheezing episodes per year (aged 3+ years)		5	[13]	
Relative Risks by age for Physician Confirmed Wheezing after Hospitalization for RSV				
	0–5	6–11	12–23	
	months	months	months	
BPD,CHD,CLD	1.25	1.25	1.25	assumed as for 29–32 weeks
<29	1.29	1.29	1.29	[68]
29–32	1.25	1.25	1.25	[68]
33–36	1.21	1.21	1.21	[68, 73]
No risk	1.24	1.24	1.13	[68, 70, 73]
	2–3	4–5	6–12	
	years	years	years	
BPD,CHD,CLD	1.14	1.03	1.00	assumed as for 29–32 weeks
<29	1.15	1.03	1.00	
29–32	1.14	1.03	1.00	
33–36	1.08	1.04	1.001	
No risk	1.07	1.06	1.04	
	[46, 68, 82–84]	[68, 73]	[68, 73]	[age-specific]
Demographic				
Average Population (2015)		7,978,067		[2]
Live Births (2015)		178,723		[2]
Disability weights				
Infants aged 0–11 months		0.00675		[a]
Infants aged 12–23 months		0.00770		[a]
Pre-Hospital Phase		0.17		[14]
Pre-Hospital Phase (caregiver)		0.03		[14]
Ambulatory Visit (included in pre-hospital)		0		
ER visit (included in pre-hospital)		0		
Days in Hospital		0.40		[14]
Days in Hospital (caregiver)		0.04		[14]
Post-Hospital Phase		0.09		[14]
Post-Hospital Phase (caregiver)		0.01		[14]
Out-patient visit (included in post-hospital)		0		
% asthma controlled or partly controlled		50%		[84]
Asthma (included after chronic phase)		0.018		[84]
Wheezing (included after chronic phase)		0.0018	per episode	[13]
Chronic Phase		0.011		[14]
Duration of Disability				
Pre-Hospital Phase	days	3.5		[57]
Post-Hospital phase	days	60		[14]
Chronic Phase	days	122		[14, 19]
Asthmatic	days per year	365		Assumed
Economic				
Exchange rate 2015	NIS per USD	3.89		[4]
Discount Rate		3%		Standard practice.
GDP per Capita 2015		$35,341		[4]
Cost-effectiveness threshold		$105,986		[5]
Unit Costs				
Ambulatory Physician	per visit	$12.83		[85]
Emergency Room	per visit	$209		[86]
General Hospital Ward	per day	$526		[86]
ICU to Pediatric Ward Cost Ratio		3.12		[17, 24, 41, 87]
Out-Patient Department	per visit	$72		[85]
Asthma - aged 0–5	per year	$1147		[12]
Asthma - aged 6–17	per year	$1311		[12]
Asthma - aged 18+	per year	$3200		[12]
Wheezing	per year	$1089		[13]
Mortality	per death	$4690		Local Burial prices
Immunoprophylaxis Costs				
Immunoprophylaxis Cost-50 mg vial		$520		[d]
Immunoprophylaxis Cost-100 mg vial		$957		[d]
Immunoprophylaxis cost per dose for 0–5 months infant		$1054		(d,[16])
Immunoprophylaxis cost per dose for 6–11 months infant		$1378		(d,[16])
Immunoprophylaxis cost per dose for 12–23 months infant		$1628		(d,[16])
Average cost per Immunoprophylaxis course		$6281		Derived from Model
Immunoprophylaxis wastage		3.3%		[7]
Hospital Doctors Costs	per hour	$41		[85]
Nurses Empoyment Costs	per hour	$27		[85]
Secretarial Costs	per hour	$15		[85]
MD time per Immunoprophylaxis dose	mins	6.1		[85]
Nurses time per Immunoprophylaxis dose	mins	30		[15, 17]
Secretarial time per Immunoprophylaxis dose	mins	3.0		Estimated
Average No. of Immunoprophylaxis shots:BPD		4.87		[61]
Average No. of Immunoprophylaxis shots: non-BPD		4.93		[6]
Caregiver Work Losses				
Average gross wage costs	USD per hour	13.26		[4]
Social overheads as % gross wage		25%		[e]
Work hours per day	hours	7.18		[4]
Time off work per vaccination	hours	4		Approximation
Time off/attack in uncontrolled asthmatics	hours	10.77		Assumed 1–2 days
Time off per severe wheezing Episode	hours	10.77		Assumed 1–2 days
	0–5	6–11	12–23	
	months	months	months	
% mothers working full time before pregnancy	45%	45%	45%	[4]
% on maternity leave	67%	10%	0%	[4]
% taking time of work for caring for sick child	15%	41%	45%	[4]
Length of work absence (non-hospitalized) days	5.9	5.3	5.5	Assumed 50% of hospitalized cases
Length of work absence (hospitalized) days	11.8	10.7	11.0	[f]
Notes:				
a) Calculations by Gary Ginsberg on HALE [4, 88]				
b) Department of Information. Ministry of Health.				
c) ICD 9 codes 017.4, 053.2, 381.0–381.4, 382.0				
d) MOH prices excluding VAT				
e) Average employers contribution to pension, health care and national insurance.				
f) Pre-hospital plus twice Average Length Of Stay				

## Appendix 2

### Cost-Effectiveness and Cost-Containment Studies

#### Cost-effectiveness Studies

- Passive immunoprophylaxis is not cost-effective.

Our study’s finding that passive immunization of RSV is not cost-effective affirms the findings of many other studies in infants with CHD [17], CLD [16], 26–32 GAW non CLD [18], 32–44 GAW [19], < 33 GAW [16, 20], < 33 GAW with > 27 days in Neonatal Intensive Care Unit, discharged between December and August [20], 32–35 GAW with less than two AAP2006 risk factors [19.21], 33–36 GAW [20] and Innuit ethnicity living in low-risk urban areas regardless of GAW [15].

b) Passive immunoprophylaxis is cost-effective.

There are many studies which reported that immunoprophylaxis was cost-effective in BPD [26, 27, 29–31], CHD [26–29, 31] < 29 GAW [24, 32], 29–32 GAW [32], < 33 GAW and > 27 days in the Neonatal Intensive Care Unit, discharged between September and November [20], < 32 GAW [19, 21],32–34 GAW with risk factors [21], 32–35 GAW with risk factors [19, 21–23], < 33 GAW [25, 29, 31], 33–35 GAW [29–31], < 34 GAW [30], < 36 GAW (ie: all preterm) [26, 27, 29, 31], < 36 GAW with risk factors [19] and Innuit heritage living in rural and high-risk urban areas regardless of GAW [15].

#### Cost-Containment Studies

c) Passive immunoprophylaxis is cost-saving

Palivisumab was actually found to be cost-saving and added QALYs among infants < 32 GAW and under 6 months old [19, 21]. Three other industry funded studies [34–36] reported a wide range of net costs, which included cost-savings. Another industry study suggested there will be net cost savings if infants under six months old living in rural or high risk Arctic Canadian communities received palivisumab [37]. A lone publicly funded study [33] showed cost savings would occur in CLD patients who received oxygen in their home setting.

d) Immunoprophylactic intervention costs exceeded savings in hospitalization costs.

A lone industry funded study with a non-directional grant [38] and many publically funded economic studies [16, 17, 39–49], reported that Palivizumab infant immunoprophylactic costs exceeded the resultant savings in hospitalization costs due to decreased morbidity.

## Abbreviations

AAP: American Academy of Pediatrics
AB: Acute Bronchitis
CHD: Congenital Heart Disease
CLD: Congenital Lung Disease
DALY: Disability-adjusted life year(s)
DW: Disability Weights
GAW: Gestational Age in Weeks
GDP: Gross Domestic Product
HALE: Healthy Adjusted Life expectancy
NHS: National Health Service (UK)
RSV: Respiratory Syncytial virus
USD: United States Dollars
